# The Influence of COVID-19 on Irrational Consumption Behavior in a Chinese Sample: Based on a Serial Mediating Model

**DOI:** 10.3389/fpsyg.2021.718797

**Published:** 2021-10-25

**Authors:** Hu Yue-Qian, Xie Piao, Wang Ying, Huang Zhi-Xin, Wu Yi-Ting, Sun Hai-Long

**Affiliations:** School of Business, Guangdong University of Foreign Studies, Guangzhou, China

**Keywords:** COVID-19, public health emergencies, perceived scarcity, negative mentality, irrational consumption behavior

## Abstract

Based on the scarcity theory, this study focuses on exploring the relationship between the severity of public health emergencies (i.e., COVID-19) and individual irrational consumer behaviors through the serial mediating variables of perceived scarcity (PS) and negative mentality (NM). An online questionnaire was used to collect data from participants in China and we obtained 466 effective (115 male and 351 female) questionnaires in total. The findings showed that the relationship between each pair of factors – perceived pandemic severity, PS, NM, and irrational consumption behaviors – was significantly positive. Although the perception of the severity of this public health emergency did not directly predict irrational consumer behavior, the effect was mediated by PS and NM independently and serially. These findings reveal that people who strongly perceive scarcity and are prone to negative attitudes are more likely to demonstrate irrational consumer behaviors (such as rushing to buy and hoard living supplies) once the public perceives a public health emergency as severe. This effect occurs because the PS that results from the epidemic affects people’s cognition, emotion, and behavior.

## Introduction

Since 2020, COVID-19 has ravaged the world. Although countries have taken measures to control its spread, this public health emergency, with its characteristics of suddenness and dangerousness, continues to exert an important impact on individual psychology and behavior (Qin et al., 2021). For example, people’s indoor time generally increases during the epidemic, so screen time increases with it. Excessive screen time use has been associated with a range of negative mental health outcomes (Lee et al., 2020). Besides, public health emergencies are often associated with different levels of irrational consumption behavior (ICB), such as the “panic buying of masks and food” in China and the “panic buying of toilet paper” in Japan, Australia, and the United States. ICB refers to unreasonable consumption decisions made by consumers under the influence of various factors which are composed of impulse and blind consumption behavior (Li and Yang, 2016). In general, previous studies have primarily examined the ICB of individuals in general situations and have explored the causes of these ICBs through four main lenses: the personal characteristics of consumption, purchase motivation, personal resources, and marketing stimulus (Verhagen and Dolen, 2011; Thompson and Prendergast, 2015; Iyer et al., 2020). However, when individuals are exposed to health emergencies, especially those related to their own health, their psychological perception, and consumption behavior may differ. For example, recent studies have pointed out that the public’s perceived scarcity (PS) is magnified in emergencies, which can also affect people’s cognition and behavior to a certain extent (Liu and Wang, 2020).

However, in the context of health emergencies, a clear explanation remains lacking of the impact of individual psychological perception and consumption behavior, as well as the transmission mechanism of psychological factors, such as PS, on ICB. Thus, this study aimed to investigate the mechanism through which the perceived severity of COVID-19 influences individuals’ ICBs.

## Theoretical Framework and Hypotheses Development

### Perceived Severity of Pandemics and Irrational Consumption Behavior

Irrational consumption behavior refers to the irrational purchase decision made by consumers when they are affected by various factors (Xinhui and Han, 2016). In a specific performance of ICB, it can also be characterized by impulsiveness (i.e., unconsidered purchase behavior or sudden purchase intention) and blindness (i.e., purchase intention caused by following trends or being influenced by the outside world; Tversky and Kahneman, 1974). The panic buying behavior is a manifestation of ICB (Asiah et al., 2021). A previous study postulated a causative model of panic buying, mentioning that there were primary, secondary, and tertiary factors stimulating this behavior (Arafat et al., 2021). The external environment affects consumers’ emotions, thereby stimulating their ICB (Chen and Yao, 2018). In public emergencies, due to concern over their severity, the public often experiences a lack of security, which leads to the social panic mentality and the panic buying of particular products, such as food, medicine, and toilet paper. For example, the panic buying phenomena during COVID-19 was caused by a combination of reasons, including PS, increased demand, necessary goods, anticipated price hike, and other responsible factors (Arafat et al., 2020). The COVID-19 pandemic is a form of public emergency, i.e., a kind of public event that happens suddenly and causes or may cause serious social harm. Public emergencies are characterized by abruptness, derivatization, harmfulness, and unpredictability (Wang et al., 2019). Some studies have pointed out that in the case of emergencies, due to supply shortages and social learning, consumers’ purchasing decisions are usually influenced by their peers’ choices, which results in greater panic among consumers and large-scale panic buying (Zheng et al., 2020). Therefore, this research proposes the following hypothesis:

*Hypothesis 1*: Perceived pandemic severity positively predicts irrational consumption behavior.

### Perceived Scarcity and Negative Mentality as Mediators

Consumers rely on many resources for survival, including those that take the form of capital or production inputs, such as time and money (Roux et al., 2015), as well as physical resources, such as food, water, and gas (Lene and Bang, 2013; Ravi and Meng, 2016). People experience resource scarcity when they perceive that the resources they possess are insufficient to satisfy their needs and desires, resulting in a series of psychological and behavioral responses. As a universal phenomenon, scarcity plays an important role in individual emotion, perception, and behavior (Fan et al., 2019). Individuals’ preoccupation with resource scarcity causes a comprehensive decline in cognition and judgment. For example, Eldar and Sendhil (2015) found that the scarcity of resources (such as time or money) fosters a scarce mindset, resulting in the loss of “bandwidth” needed for decision making. According to the scarcity theory, the individual’s attention is unconsciously occupied in the state of scarcity, which reduces the amount of cognitive “bandwidth” to invest in other things, leading to declines in one’s computing, concentration, cognitive, planning, and self-control capacities. Some studies in the consumer field have also shown that the scarcity of goods exerts an impact on consumers’ perception of commodity value, which will promote their impulse to consume products (Jang et al., 2015). Based on the scarcity theory, we propose the following:

*Hypothesis 2*: Individual perceived scarcity mediates the relationship between perceived pandemic severity and irrational consumption behavior.

Emotions can guide people to make decisions under conditions of risk and uncertainty that involve intertemporal choice, social decision making, and ethical decision making. One study found that, in response to the COVID-19 pandemic, 66.4% of the respondents living in Addis Ababa, Ethiopia, experienced moderate to severe psychological problems, including stress, anxiety, and depression (Kassaw, 2020). During this emergency, people are faced with danger, and social emotions will alter their behaviors to a certain extent. A previous study has shown that emotion is one of the most important factors in the irrational decision-making process. Especially under the interference of negative social mentality, people’s negative emotion will affect their own decision making (Lerner et al., 2013).

Social mentality is the general social attitude, emotional experience, intention, and other psychological states that people hold toward themselves and their society. This mentality is formed on the basis of the convergence, integration, mutual influence, and mutual infection of different individual psychologies (Wang and Ying, 2020). Social mentality has a strong inductive effect on people’s social behavior and is an important psychological basis for social disorder. Changes in panic and anxiety are the most prominent experience and feeling in public health emergencies (Wang and Ying, 2020). One study presented that almost 30% of Mexican individuals presented with clinically significant symptoms of posttraumatic stress during the COVID-19 pandemic, and most people in quarantine experienced negative emotions; moreover, many people engaged in irrational behavior, such as panic purchasing, discriminatory behavior, and violence against health professionals (Ramírez et al., 2020). In times of emergency and crisis, due to the resulting environment of fear and insecurity, people will indulge in the behaviors necessary for survival. Furthermore, sensationalized media reporting on the crisis can also trigger negative emotions, such as panic and anxiety, which will lead to more panic buying (Arafat et al., 2020).

Therefore, we believe that public anxiety, panic, and other examples of negative mentality (NM) caused by public health emergencies can affect individual ICB. Based on the abovementioned research, this study proposes the following:

*Hypothesis 3*: Negative mentality mediates the relationship between perceived pandemic severity and irrational consumption behavior.

According to scarcity theory, PS will change people’s mode of thinking and will affect their decision making and behavior. Previous studies have shown that PS exerts an impact on individual cognitive ability and executive control (Arafat et al., 2020). In addition, the scarcity perceived by the public can be seen as a feeling of losing control, which will lead to panic behavior and vastly increased hoarding behaviors (Zheng et al., 2020). Such responses may also occur to reduce the fear and anxiety caused by the loss of control over one’s surrounding environment during the pandemic. Therefore, we believe that in the face of public health emergencies, due to the expected lack of access to products or services and the prevalence of NM, the public will demonstrate a strong demand for products, which leads to a sense of urgency to buy and hoard an excessive amount of merchandize. Therefore, this research proposes the following:

*Hypothesis 4*: Perceived scarcity and negative mentality operate as serial mediators between perceived pandemic severity and irrational consumption behavior.

## Study Methods

### Participants

This cross-sectional multicenter study was conducted from February 22 to March 17, 2020. According to statistics from the National Health Commission, the total number of confirmed COVID-19 cases in mainland China increased from 76,936 to 82,798 during this period. The investigated sample was recruited online *via* Wenjuanwang,^1^ an online platform similar to Mechanical Turk or Qualtrics that is used to launch nationwide e-surveys in China and is widely employed in behavioral and psychological studies. Participants gave their informed consent after being provided with information explicitly stating the research purpose, nature, and procedure of the study. A total of 525 questionnaires were collected. After excluding the ones with missing values, 466 valid data were obtained, with an efficiency rate of 88%. The average participant age is 23.6years (*SD*=7.4). Table 1 presents the demographic characteristics of the participants.

**Table 1:** Demographic characteristics of the sample.

Variable	Category	Frequency (*N*)	Percentage (%)
Gender	Male	115	24.7
	Female	351	75.3
Age	18–25	383	82.2
	≥26	77	17.8
Education status	High school or below	95	20.3
	Bachelor degree	371	79.7
Family income	< ¥24,000	145	31.1
	¥24,000–¥60,000	126	27
	>¥60,001	195	41.9

### Measures

The measures used were identical to those that have been widely used in previous studies and were translated into Chinese, ensuring that the scale can be understood by respondents in China.

#### Perceived Pandemic Severity

The existing and internationally accepted definition of a pandemic, as provided by the WHO and the Dictionary of Epidemiology, comprises three dimensions: mortality, morbidity, and comorbidities (Campos, 2020). Therefore, perceived pandemic severity (PPS) was measured through three dimensions: the number of casualties, the scope of spread, and the duration. Each item is rated from 1 (strongly disagree) to 5 (strongly agree), with higher scores indicating higher perceived severity. Moreover, the PPS in the current study included country-specific questions (such as: From the perspective of the number of confirmed cases and deaths, do you think this outbreak is serious?”; *α*=0.83).

#### Perceived Scarcity

Perceived scarcity was measured by a 5-item scale developed by Roux et al. (2015). The items are rated on a 5-point scale ranging from 1 (strongly disagree) to 5 (strongly agree). Higher scores indicate higher perceptions of scarcity (indicated by questions, such as “My materials are insufficient”; PS: *α*=0.86).

#### Negative Mentality

The NM instrument was revised and adapted from Wang’s research on SARS (Wang et al., 2003). The scale consists of eight items, each rated on a scale from 1 to 5 (1=never and 5=very often). Higher scores indicate a more NM (indicated by questions, such as “I felt nervous and afraid due to the epidemic situation”). The subscales indicated good internal consistency in this sample (NM: *α*=0.87).

#### Irrational Consumption Behavior

The level of individual consumption behavior was measured with a questionnaire originally developed by Kahneman (Xinhui and Han, 2016) that includes four items. The scale comprises two subscales: impulsiveness (indicated by questions, such as “I will stock up on materials in large quantities”) and blindness (e.g., “If the ingredients in a certain drug can prevent new pneumonia, I will buy it even if its messaging is inaccurate”). Each item is rated from 1 (strongly disagree) to 7 (strongly agree), with higher scores indicating higher ICB. The total score of this scale was used in the analysis. The scale demonstrated good internal consistency in this sample (ICB: *α*=0.83).

#### Control Variables

Some previous studies have suggested that gender and age may influence ICB; therefore, we controlled for gender and age in the data analysis (Wang et al., 2018). ICB has also been shown to correlate with income (Pati et al., 2020), so we also collected the data on annual household income as an objective indicator.

### Data Analysis

All data were analyzed with IBM SPSS 25.0 for correlation, reliability, regression, and confirmatory factor analyses. We used Amos for SPSS to conduct confirmatory factor analyses and Hayes’ PROCESS macro for SPSS to test the proposed serial mediation model.

## Results

### Control of Common Method Biases

First, an anonymous online survey was adopted and questionnaires were distributed and collected at different times, which, in part, controlled common method biases. In addition, Harman’s one-factor test was used to ensure statistical control. Exploratory factor analysis was used to examine the four variables. Therefore, the Harman single-factor analysis method was adopted in this study to test all variables, and principal component analysis was conducted. The results shown that the cumulative explanation rate was 63.13%, and there were four factors with eigenvalues greater than 1. The first common factor explains 32.28% of the total variance, which does not exceed 40%. Therefore, no serious problem exists among common method biases.

### Measurement Model

Before the theoretical model was tested, confirmatory factor analyses (*CFA*) were performed with Amos to examine the discriminant validity of four key variables. As shown in Table 2, the proposed four-factor model (PPS; PS; NM; and ICB) revealed an acceptable fit (Model 1): (χ^2^/*df*=3.22, *CFI*=0.92, *TLI*=0.91, *RMSEA*=0.07) and fit better than alternative models (Models 2 to 4). The results showed significant discriminant validity for the four key variables. Furthermore, we examined the average variance extracted (*AVE*) and composite reliability (*CR*) of each variable: PPS (*AVE*=0.62, CR=0.8273), PS (*AVE*=0.5572, CR=0.8577), NM (*AVE*=0.46, CR=0.8729), and ICB (*AVE*=0.5994, *CR*=0.8614). The *AVE* values of variables, except for NM, are larger than 0.5, and the *CR* of all variables is over 0.8.

**Table 2:** Comparison of measurement models.

Model	Description	*χ* ^2^	*df*	*χ* ^2^ */df*	RMSEA	CFI	TLI
Model 1	Four factors: PPS, PS, NM, ICB	528.44	164	3.22	0.07	0.92	0.91
Model 2	Three factors: PPS+PS, NM, ICB	1060.93	167	6.35	0.11	0.80	0.77
Model 3	Two factors: PPS+PS+NM, ICP	1801.87	169	10.66	0.14	0.63	0.59
Model 4	One factor: PPS+PS+NM+ICP	2362.98	170	13.90	0.17	0.51	0.45

*N=466. PPS, perceived pandemic severity; PS, perceived scarcity; NM, negative mentality; ICB, irrational consumption behavior; RMSEA, root mean square error of approximation; CFI, comparative fit index; and TLI, Tucker-Lewis Index*.

### Correlations and Descriptive Statistics

Table 3 shows the descriptive statistics and correlations among the research variables. The results show that PPS is positively correlated with PS (*r*=0.138, *p* < 0.01), NM (*r*=0.287, *p* < 0.01), and ICB (*r*=0.118, *p* < 0.05). PS is positively correlated with NM (*r*=0.391, *p* < 0.01) and ICB (*r*=0.292, *p* < 0.01). The results also indicated that NM is positively correlated with ICB (*r*=0.496, *p* < 0.01).

**Table 3:** Descriptive statistics and correlations.

S. No.		*M*	*SD*	1	2	3	4	5	6	7	8
1.	Gender	1.75	0.43								
2.	Age	22.49	7.35	−0.105^*^							
3.	Occupation	2.93	0.55	0.053	−0.239^**^						
4.	Education status	4.69	0.70	−0.059	−0.206^**^	−0.136^**^					
5.	Family income	4.26	1.06	−0.040	0.024	−0.104^*^	0.071				
6.	PPS	4.56	0.56	0.151^**^	0.019	−0.008	0.093^*^	−0.003			
7.	PS	3.42	1.08	0.069	0.081	−0.014	0.122^**^	−0.042	0.138^**^		
8.	NM	2.98	0.91	0.158^**^	0.148^**^	0.015	−0.023	−0.007	0.287^**^	0.391^**^	
9.	ICB	2.11	0.93	0.046	0.140^**^	0.039	−0.039	−0.015	0.118^*^	0.292^**^	0.496^**^

* *p<0.05;*

** *p<0.01;*

*N=466*.

*PPS, perceived pandemic severity; PS, perceived scarcity; NM, negative mentality; and ICB, irrational consumption behavior*.

### Serial Multiple Mediation Analyses

We used model 6 of the PROCESS macro with 5,000 resamples to conduct a serial mediation analysis. We treated PPS as the independent variable (X), PS as the first mediator (M1), NM as the second mediator (M2), and ICB as the dependent variable (Y). The serial mediation model contains one direct effect (X→Y) and three indirect effects (Ind1: X→M1→M2, Ind2: X→M2→Y, and Ind3: X→M1→M2→Y).

#### Direct Effect Testing

According to the results, Hypothesis 1, which predicts a positive relationship between PPS and ICB, is not supported (*β*=−0.049, SE=0.069, *p*=0.479>0.05).

#### Mediating Effects Testing

**Figure 1:**
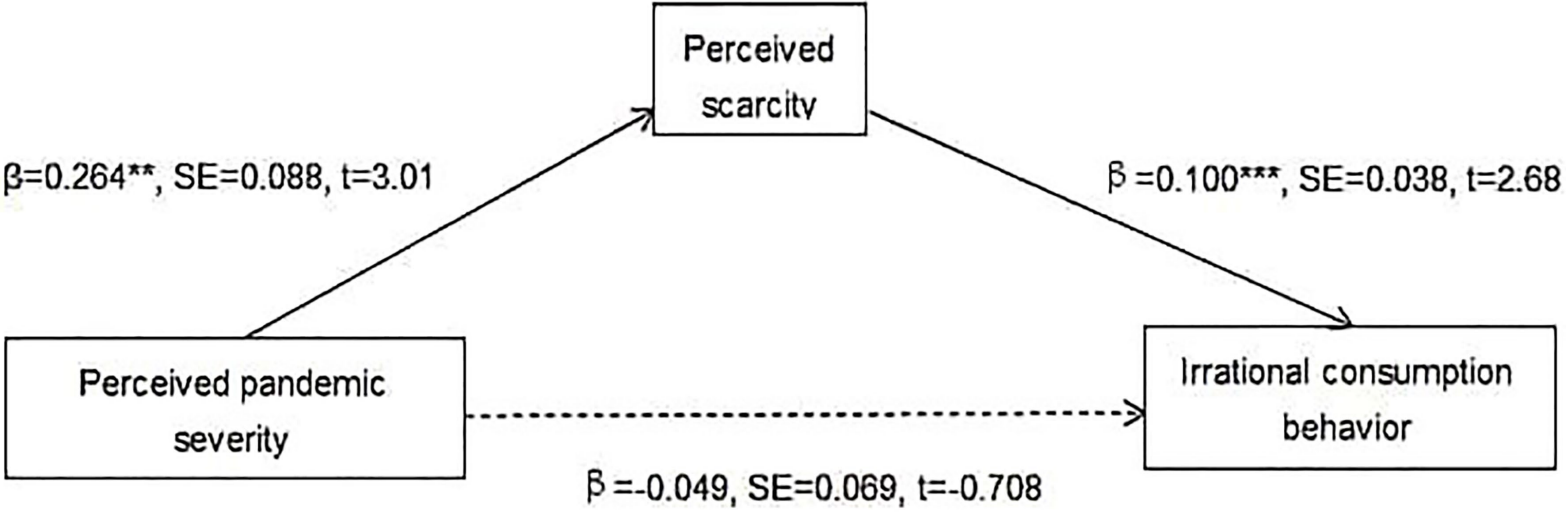
Mediation effect of perceived scarcity (PS). ^**^p<0.01; and ^***^p<0.001; full lines indicate significant paths; and dashed line indicates a nonsignificant path.

**Figure 2:**
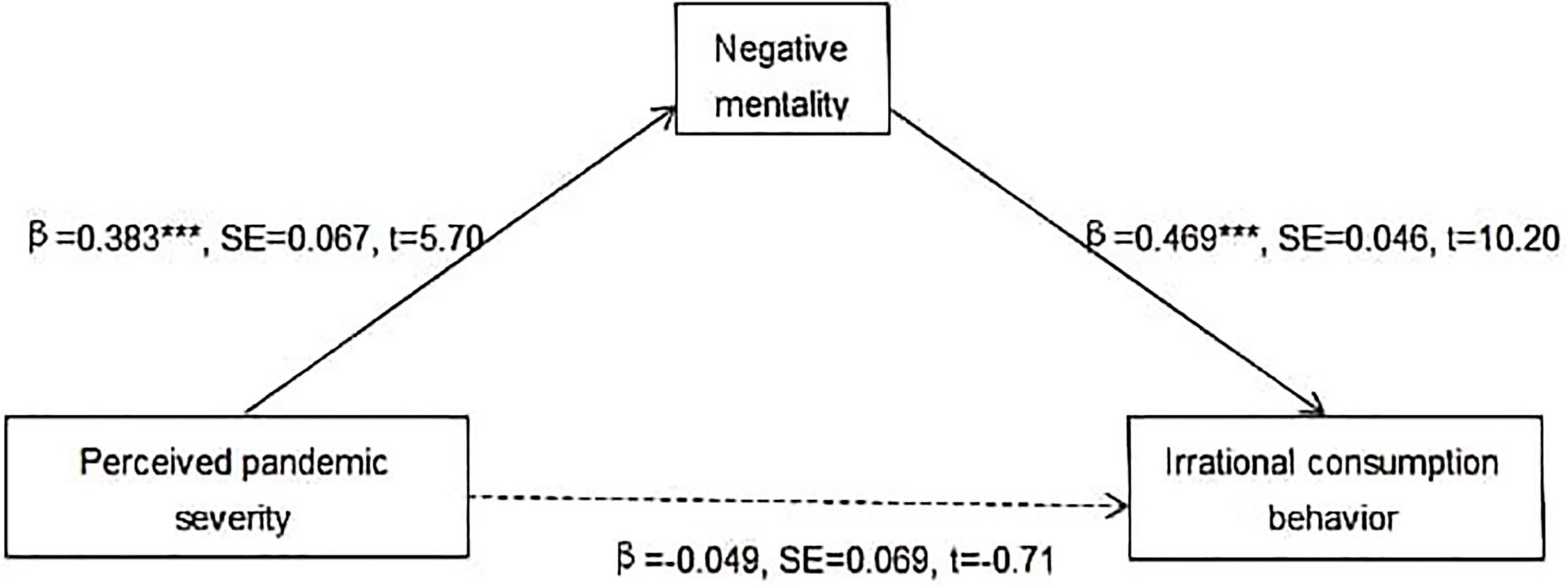
Mediation effect of negative mentality (NM). ^***^p<0.001; full lines indicate significant paths; and dashed line indicates a nonsignificant path.

First, as presented in Figure 1, the results suggest that the indirect effect of PPS on individuals’ ICB through PS (Ind1: X→M1→M2) is significant [*b*=0.27, boot SE=0.015, 95% CI=(0.0045, 0.065), excludes zero]. Furthermore, as shown in Figure 2, the indirect effect of PPS on individuals’ ICB *via* NM (Ind2: X→M2→Y) is significant [*b*=0.180, boot SE=0.036, 95% CI=(0.117, 0.260), excludes zero]. The results suggest that PS and NM exercise complete mediation, respectively, between PPS and ICB. Thus, Hypotheses 2 and 3 are partly supported.

#### Serial Mediating Effect Testing

**Figure 3:**
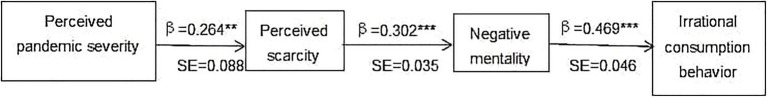
Serial mediation effects of PS and NM. ^**^p<0.01; and ^***^p<0.001; full lines indicate significant paths; and dashed line indicates a nonsignificant path.

As shown in Figure 3, the results of bootstrapping through the PROCESS macro indicate that the indirect effect of PPS on individuals’ ICB *via* PS and NM (Ind3: X→M1→M2→Y) is significant [*b*=0.037, boot SE=0.014, 95% CI=(0.014, 0.071), excludes zero]. These findings indicate that mediating effects account for all the observed relationships between PPS and ICB. Therefore, the serial mediation effect is significant, and Hypothesis 4 is partly supported.

**Figure 4:**
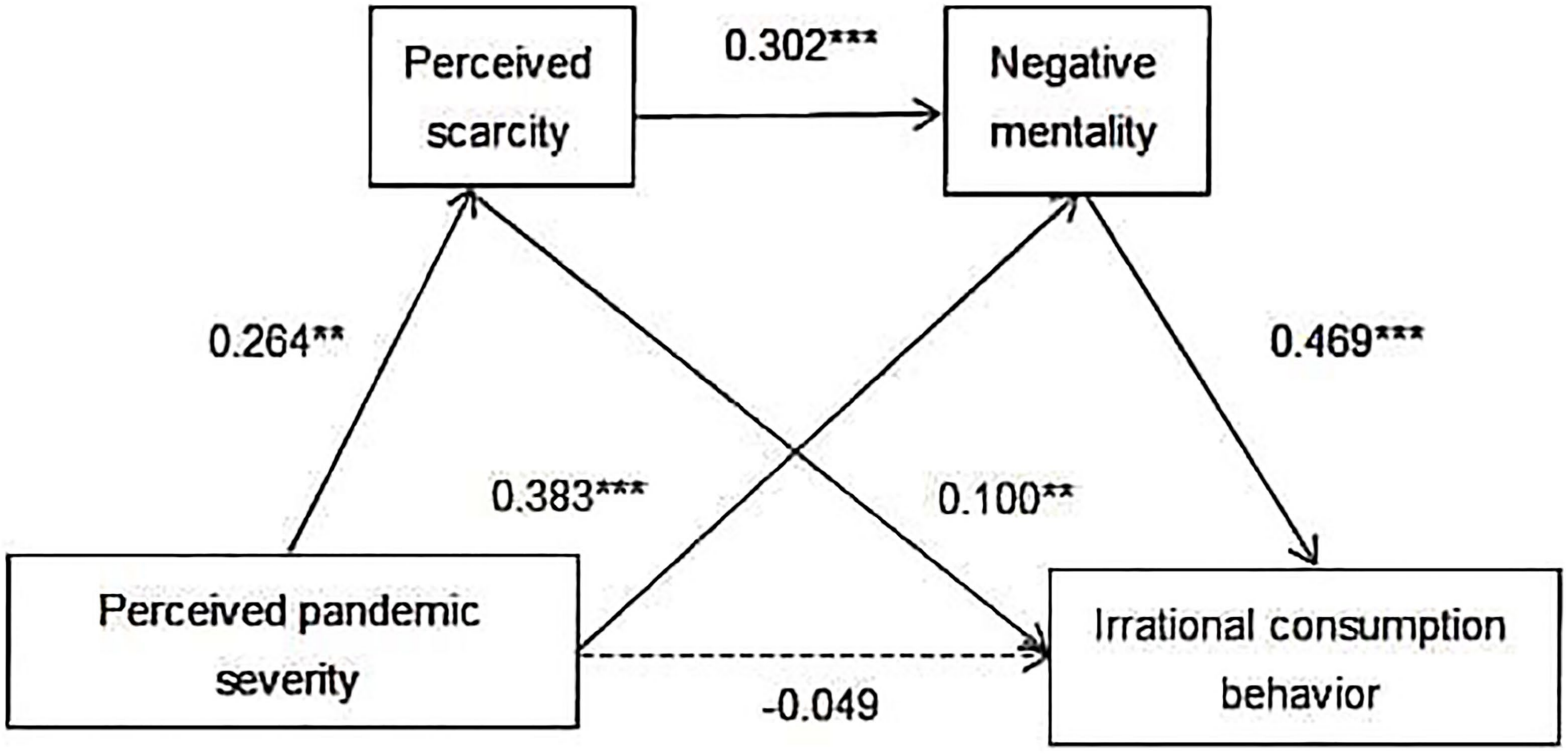
Mediation effects of PS and NM. ^**^p<0.01; and ^***^p<0.001; full lines indicate significant paths; and dashed line indicates a nonsignificant path.

Overall, as presented in Figure 4, three hypotheses in this study are supported.

## General Discussion

The current study primarily investigated the influence of PPS on irrational consumer behavior in the noninfected general public during COVID-19 and the multiple mediating effects of PS and NM. The results shown that PPS influenced irrational consumer behavior through the indirect paths of PS and NM as well as through their serial mediating path.

Consistent with the previous study and our hypotheses, when the public perceives danger from their surroundings or other places, they are more likely to be motivated by PS, which in turn increases the possibility of irrational consumer behavior. This study indicated that although PPS cannot directly predict irrational consumer behavior, it can indirectly predict such behavior through the mediating role of PS. One possible explanation is that people’s perception of pandemic severity primarily exerts a direct effect on their overall perception, which then affects their behavior through the weakening of their ability to perceive situations rather than by directly affecting people’s behavior (Zheng et al., 2020). Based on the scarcity theory, due to the decreased circulation of external resources within a short time during a pandemic and the heightened perceived uncertainty of the external environment, individuals will develop a scarcity mentality when faced with resource scarcity, consequently weakening their ability to deal with other tasks (Kim et al., 2020). Thus, such individuals seek external stimuli to reduce their feelings of insecurity and panic, particularly stimuli, such as purchases, thereby producing irrational consumer behavior and hoarding. For example, individuals in a pandemic may lose their common sense in realizing what is useful for them, instead rushing to buy useless medicine and hoard daily necessities (Jang et al., 2015).

Multiple mediation analysis further revealed several reasons why PPS leads to the public’s irrational consumer behavior. The first reason is that people perceive more scarcity during the COVID-19 pandemic. According to the scarcity theory, PS affects individuals’ thinking patterns, such as their cognitive ability, external perception, and emotions, thereby making them more prone to NM (Arafat et al., 2020). Specifically, the public tends to have relatively low perceptions of the available resources due to the disruption of COVID-19 to the resource supply and the threat to life posed by the virus (Ravi and Meng, 2016). For instance, there was a shortage of masks early in the COVID-19 pandemic. Most people who did not have masks felt their lives threatened by COVID-19, resulting in a sense of worry and a high level of fear of the external environment. Moreover, the effect of chain mediation was stronger than the unilateral mediation of PS or NM, as shown by the public’s stronger willingness to engage in panic buying and hoarding of certain goods.

Another reason is that people experience a negative mindset during the COVID-19 pandemic. NM is a kind of social mentality composed of the attitude and emotions that one holds toward oneself and social reality (Zhang et al., 2018). A NM means that the public is prone to feelings of insecurity and distrust as a result of the suddenness of the crisis within the external environment created by the epidemic (Kassaw, 2020). Individuals who are highly sensitive to the external environment are usually more susceptible to negative emotion and tend to seek external stimuli to ease such experiences during emergencies. Therefore, such individuals engaged in more irrational consumer behaviors during the epidemic (Gu and Luo, 2008). For instance, individuals received all kinds of negative information through friends or on the Internet during the pandemic that made them feel anxious and even experience trouble sleeping, which then led them to believe the various rumors that resulted in panic buying behavior as a psychological compensation for COVID-19 (Arafat et al., 2020).

## Limitations and Future Directions

Despite its contributions, this research has several limitations that leave room for future consideration.

First, all measurement scales in our study were adopted from previous studies, which reduced vague terms and item ambiguity to some extent. However, confounding variables should be addressed. For example, using questionnaires to collect data has some limitations and the possibility of causing recall bias. This study collected data from consumers during the period of COVID-19, but we could not collect data from the respondents while they were purchasing. Moreover, the data used in this study are cross-sectional, so it is still difficult to avoid common method bias by using this approach. Future research should collect data from multiple informants and adopt a more carefully controlled experimental design to investigate the relationship between the variables further.

Second, this research is limited to people from different provinces and cities in China, so it has limitations regarding cultural adaptation. COVID-19 is rampant and has a far-reaching impact worldwide. Although psychological/behavioral constructs are general, they may also be impacted by sociocultural factors. The results can therefore not be generalized to the population as a whole. Thus, in the future, data from different countries can be collected for further study, and culture-related variables can be introduced to conduct cross-cultural studies on individuals’ ICBs in different countries and regions. For instance, Jovančević and Milićević carried out a cross-cultural study to examine the factors (e.g., optimism and pessimism) causing COVID-19-related behaviors (e.g., hoarding and preventive behaviors) in Serbia and Latin America (Jovančević and Milićević, 2020).

Third, this study focuses on researching individual ICB from the perspective of scarcity theory within emergent public health events. Although we believe that the scarcity mindset represents an important potential mechanism behind ICB, its mechanism of influence on public health emergencies can be evaluated from other perspectives. For example, individual differences might modulate the relationship between public health emergencies and their associated behavioral consequences. Shahjehan and Qureshi (2019) examined impulsive buying behaviors through the Big Five personality traits and found that people engaged in different levels of impulsive buying behavior had different personality traits (e.g., conscientiousness and neuroticism). Moreover, the impact of COVID-19 on individuals’ ICB is a dynamic process (Franch-Pardo et al., 2020). The ICB of individuals will change with the intensity of COVID-19 and the government’s policy guidance. For example, Oni et al. (2020) found that governmental interventions influenced residents’ diet and physical activity behavior, as well as their buying behavior. Thus, subsequent studies should focus on additional factors, such as the governmental interventions and government’s credibility (Bonneux and Van, 2006) to identify other potential mechanisms that influence individual ICB (Coninck et al., 2020).

## Conclusion

The COVID-19 pandemic has caused changes in consumer behavior. Our study highlights the relationship between the perceived severity of pandemics and ICB. Through the application of scarcity theory, the current study proposes a model that elucidates the relationship between PPS and consumers’ irrational consumer behavior.

To summarize, the results show that as: (1) Individual PS mediates the relationship between PPS and ICB. (2) NM mediates the relationship between PPS and ICB. (3) PS and NM operate as serial mediators between PPS and ICB.

## Data Availability Statement

The original contributions presented in the study are included in the article/Supplementary Material, further inquiries can be directed to the corresponding author.

## Ethics Statement

The studies involving human participants were reviewed and approved by the ethical standards of institutional review board at Guangdong University of Foreign Studies. The patients/participants provided their written informed consent to participate in this study.

## Author Contributions

SH-L and HY-Q conceived and designed the experiments. HZ-X and WY-T performed the experiments. HY-Q and XP analyzed the data. SH-L and WY contributed to reagents, materials, and analysis tools. HZ-X and WY-T wrote the paper. All authors contributed to the article and approved the submitted version.

## Funding

This research was partially supported by the GuangDong Basic and Applied Basic Research Foundation (no. 2020A1515110429), the Youth Foundation of Social Science and Humanity, China Ministry of Education (no. 20YJCZH135), the Youth Project of Social Science Foundation of Guangdong Province (no. GD19YGL07), and the Undergraduate training program for innovation and entrepreneurship of Guangdong province (no. S202011846034).

## Conflict of Interest

The authors declare that the research was conducted in the absence of any commercial or financial relationships that could be construed as a potential conflict of interest.

## Publisher’s Note

All claims expressed in this article are solely those of the authors and do not necessarily represent those of their affiliated organizations, or those of the publisher, the editors and the reviewers. Any product that may be evaluated in this article, or claim that may be made by its manufacturer, is not guaranteed or endorsed by the publisher.

## Footnotes

^1^ http://www.wenjuan.com
